# DoE-Assisted Development of a Novel Glycosaminoglycan-Based Injectable Formulation for Viscosupplementation

**DOI:** 10.3390/pharmaceutics12070681

**Published:** 2020-07-20

**Authors:** Marta Cicognani, Silvia Rossi, Gabriele Vecchi, Andrea Maria Giori, Franca Ferrari

**Affiliations:** 1 R&D Department, IBSA Farmaceutici Italia Srl, Via Martiri di Cefalonia 2, 26900 Lodi, Italy; marta.cicognani@ibsa.it (M.C.); gabriele.vecchi@ibsa.it (G.V.); andrea.giori@ibsa.it (A.M.G.); 2 Department of Drug Sciences, University of Pavia, Viale Taramelli, 12, 27100 Pavia, Italy; franca.ferrari@unipv.it

**Keywords:** hyaluronic acid, viscoelastic injectable solutions, viscosupplementation, DoE approach, phosphatidylcholine, tribology measurements

## Abstract

The aim of the present work was the development of a novel glycosaminoglycan (GAG)-based injectable formulation intended for intra-articular administration that should best mimic the healthy synovial fluid. Hyaluronic acid (HA) was chosen among GAG polymers, since it is the most abundant component of the synovial fluid. A DoE (Design of Experiment) approach was used for the development of a formulation containing two HA (very high (VHMW) and low (LMW) molecular weight) grades. The rationale for this choice is that so far, no commercial product based on a single HA grade or even on binary HA mixture possesses optimal viscoelastic properties in comparison with healthy synovial fluid. A full factorial design was chosen to investigate the influence of concentration and relative fraction of the two polymer grades (retained as factors of the model) on formulation functional (viscosity and viscoelastic) properties, which are considered response variables. Thanks to the DoE approach, the composition of the optimized HA formulation was found. The addition to such formulation of an injectable grade fat-free soy phospholipid, which was rich in phosphatidylcholine (PC), resulted in improved lubrication properties. The final HA + PC formulation, packaged in pre-filled sterile syringes, was stable in long-term and accelerated ICH (International Council for Harmonisation) storage conditions. The overall results pointed out the formulation suitability for further steps of pharmaceutical developments, namely for the passage to pilot scale.

## 1. Introduction

All human joints are lined with a tissue called synovium, which is surrounded by synovial fluid [1]. This is a viscous, non-Newtonian fluid, whose main role is to reduce frictions between the articular cartilages during movements. Synovial fluid contains glucose, uric acid, proteins (lubricin), proteoglycan, polysaccharides (in particular glycosaminoglycans), and phospholipids [2].

Hyaluronic acid (HA), also known as hyaluronan, is the major component of synovial fluid and its concentration in healthy joints is 1.45–3.12 g/L [3].

HA is a high-molecular-weight linear (MW) natural glycosaminoglycan, consisting of D-glucuronic acid and D-N-acetylglucosamine disaccharides bound with β-glycosidic linkages [4]. It is an essential component of the extracellular matrix (ECM) that is present at high concentration in human umbilical cord, synovial fluids, connective tissue, skin, and vitreous body [5]. Since carboxyl groups (COO–) are completely ionized at physiological pH, it possesses hydrophilic and water-retaining properties, forming a viscous gel that is characterized by peculiar rheological properties. In particular, HA solutions are characterized by a gel-like behaviour at high frequencies and a liquid-like behaviour at low frequencies. In physiological fluids, the ions shield the negative charges of polymer backbone, and HA chains behave as random coils. The entanglement concentration decreases on increasing MW, meaning that the longer chains overlap each other at lower concentration. An increase in MW and/or concentration corresponds to high values of viscoelastic moduli and a marked shear-thinning behaviour [6]. These properties are relevant for the structural function of HA in connective tissue as well as for the lubricating and cushioning activities in the ophthalmic aqueous humour and synovial fluid [4].

Chemically, hyaluronan is a linear high MW polysaccharide consisting of repeating units of D-glucuronic acid and N-acetyl-glucosamine, with an average molecular weight of 7 × 10^6^ g/mol in healthy synovial fluid [5].

Phospholipids (i.e., phosphatidylcholine) are small amphiphilic molecules that are present in synovial joints at a relatively high concentration, 0.1–0.2 mg/mL [7].

The synovial fluid of joints normally functions as a biological lubricant, providing low-friction and low-wear properties to articulating cartilage surfaces through the putative contributions of HA and surface active phospholipids [2].

In order to perform this function, the synovial fluid has a viscoelastic, non-Newtonian, behavior because its viscosity varies with the variation of the applied force. In the absence of movement, or with slow movements, the viscous behavior prevails (measured by the viscous modulus G’’) and therefore the lubrication capacity increases as well.

When joints are in movement (for example during rapid walking or running), elastic behavior prevails (measured by the elastic modulus G’), which is essential to protect joints during loading.

In the synovial fluid of a healthy individual, the inversion between the two modules occurs at low frequencies, generally lower than 1–2 Hz. This frequency, called the cross-over frequency, corresponds for example to the transition from slow walking to running.

However, in older people, in arthritis, injury, and artificial joint failure, there is an increased friction between the joint surfaces and concomitant erosion of the load-bearing elements [8].

In these cases, the cross-over frequency is characterized by the disappearance of the cross-over point. In fact, the synovial fluid never shows a predominance of elastic behavior, and G’ is always lower than G’’. This change in the rheological properties of the fluid depends on a change in the composition. There is a reduction both in the average MW and concentration of HA.

As a further consequence, the dynamic viscosity drops, passing from an average value of some tens of Pa.s to values close to or lower than 1Pa.s. Similarly, the value of G’ at the frequency of 2.5 Hz drops from about 120 Pa to values below 10 Pa [9,10].

A variety of treatments have been proposed to restore the physiological joint environment in case of injury and arthritis. Some biological drugs proved capable of reducing pain and inflammation [11,12]. Therapeutic joint lavage has been used, as such or in combination with anti-inflammatory steroids [13,14] to cleanse the joint from cartilage degradation products, pro-inflammatory cells, and destructive enzymes associated with arthritis [15].

Injections of HA in the joint cavity (called viscosupplementation) have been reported to restore the viscosity and protective functions of the synovial fluid [16,17].

HA, usually sodium hyaluronate, is frequently used in aqueous solution for viscosupplementation. HA can be used in its native form, thus being characterized by linear molecules, or it can be chemically cross-linked to modify its aqueous solubility.

The rheological properties of HA solutions are directly influenced by the MW, concentration, and cross-linking. The higher the MW, the better the viscoelastic behavior of the solution. Unfortunately, MWs similar to the physiological ones (6–7 × 10^6^ Da) are not achieved with usual production methods; as a consequence, the hyaluronate used in viscosupplementation has an average molecular weight of 1–4 × 10^6^ Da.

To overcome this limit, one solution could be the increase of HA concentration, but this determines a viscosity increase that, in turn, can make administration difficult.

Given these premises, the aim of the present work was the development of a viscoelastic glycosaminoglycan (GAG)-based formulation in a pre-filled sterile syringe that is capable of best mimicking the functional (rheological and lubricating) properties of healthy synovial fluid when administered into the impaired joint.

The research project was divided in two steps: the first one was addressed to the DoE (Design of Experiment)-assisted optimization of a sterile injectable formulation based on two HA grades, having different MWs. Such an approach is helpful in pharmaceutical development, since it can predict the optimal composition satisfying all the formulator’s demands on the basis of few experiments and statistical analysis of the results. Therefore, thanks to this approach, the best formulation can be found on a sound mathematical and statistical basis, saving time and effort in comparison to a conventional trial and error approach [18].

The rationale for this formulation choice is based on the knowledge that, so far, no commercial products intended for viscosupplementation and based on a single HA grade or even on binary HA mixture possesses optimal viscolelastic properties in comparison with healthy synovial fluid.

The HA grades were a very high MW (VHMW) produced by fermentation, which was chosen since it represents the highest HA MW currently available, and a low MW (LMW) that was expected to reduce the dynamic viscosity at low shear rates.

HA concentrations were set in the range 1–3% w/v in order to avoid the attainment of a too-viscous system that is not comparable with the healthy synovial fluid and not compatible with industrial plants (namely syringe-filling machine due to the too high pressure required).

The influence of HA MW and concentration on formulation rheological properties was investigated. A full factorial design was employed to identify the optimal formulation in terms of concentration and relative fraction of two HA MWs, i.e., the one characterized by the best functional (viscous and viscoelastic) properties in comparison with those of healthy synovial fluid. In the second step of the work, an endogenous amphiphilic molecule (phosphatidylcholine, PC) was added to the HA-based formulation optimized in the first step in order to improve lubricant properties as well as to obtain a composition as similar as possible to that of synovial fluid, in which phospholipids represent the second major component after HA. The development was focused on the study of PC incorporation mode into the aqueous HA product, i.e., on the more suited manufacturing process. The improvement of the lubricating properties in comparison with the optimized HA formulation, a commercial product based on HA at higher concentration, and physiologic solution (0.9% w/w NaCl aqueous solution) used as control was assessed by means of tribology measures.

Tribology allows investigating the frictions of cartilages under defined conditions, both in oscillatory or rotational motion over a broad range of sliding velocities and normal pressures. Thanks to tribology measures, it is therefore possible to investigate the lubricating properties of a formulation.

The final HA + PC product, packaged in pre-filled sterile syringes, was subjected to a preliminary stability test (6 months) in ICH (International Council for Harmonisation) long-term and accelerated conditions: at each time point frequency at cross over, dynamic viscosity at 0.01 s^−1^ shear rate, pH and osmolality were compared on a statistical basis with those measured at time zero.

## 2. Materials and Methods

### 2.1. Materials

The following materials were used: injectable grade sodium hyaluronate very high MW (VHMW HA): 3.5 × 10^6^ Da (HTL Sas, Javene, France); injectable grade sodium hyaluronate low MW (LMW HA): 100,000 Da (HTL Sas, Javene, France); sodium chloride (Merck & Co., Frankfurt, Germany); dibasic sodium phosphate anhydrous (Chemische Fabrik Budenheim KG, Budenheim, Germany); monobasic sodium phosphate dehydrate (Chemische Fabrik Budenheim KG, Budenheim, Germany); water for injections (WFI) (Ph Eur. 10 Ed., Monograph 0169); injectable grade fat-free soybean phospholipid rich in phosphatidylcholine (Lipoid GmbH, Ludwigshafen, Germany).

### 2.2. Sample Preparation

Polymer solutions with total HA concentration equal to 1%, 2%, or 3% w/v were prepared by hydrating for 12 h at 50 °C the two HA grades in pH 7.0 buffered aqueous solution, obtained by adding a fixed NaCl amount (0.75% w/w) to a pH 7.4 phosphate buffer solution (0.05% dibasic sodium phosphate anhydrous, monobasic sodium phosphate dihydrate in 100 mL of distilled water). Then, the bulk solutions were divided into syringes formed by a luer lock syringe barrel 2.25 mL (BD Hypack^TM^SCF^TM^, Becton Dickinson, Le Point de Claix, France) and a plunger (West Pharmaceutical Services Inc., Exton, PA, USA).

Pre-filled syringes were steam sterilized in autoclave (mod. FOB2-TS, Fedegari, Pavia, Italy) at 121 °C for 15 min and formulations were characterized as described in Secion 2.4. The sterilization process produces a marked decrease in sample viscosity ranging from 80% to 25%, depending on the shear rate considered (greater at low shear rates).

### 2.3. DoE Approach: Full Factorial Design

A full factorial design, considering all the possible combinations between the factors and their levels, was chosen [19,20,21].

Namely, two factors (corresponding to the polymer concentration (factor A) and relative fraction of the two HA grades (factor B)) at three levels were considered; therefore, experimental trials were carried out on 3^2^ (9) possible mixtures of the two components.

For each factor, the upper level was indicated as +1 and the lower level was as indicated as –1. In particular, for the factor “polymer concentration”, the three levels were: 1% w/v (–1), 2% w/v (0), 3% w/v (+1); for the factor “relative fraction” of the two HA grades, the three levels were: VHMW:LMW HA weight ratio 1:9 (–1), VHMW:LMW HA weight ratio 5:5 (0), VHMW:LMW HA weight ratio 9:1 (+1).Therefore, each formulation was characterized by two values (Table 1): the first one (on the left) relevant to the VHMW: LMW HA weight ratio, and the second one (on the right) relevant to the HA % w/v concentration.

In Table 2, the % (w/v) compositions of the 9 formulations of the full factorial design are reported.

### 2.4. Characterization of Formulations of the Full Factorial Design

The formulations, corresponding to the different experimental points of the full factorial design, were characterized for pH (pH meter Seven Compact S220 (Mettler Toledo, Columbus, OH, USA), osmolality (Cryoscopic osmometer Osmomat O30, Gonotec, Berlin, Germany), and rheological properties (frequency and viscosity at cross-over point, tan δ and dynamic viscosity), as described hereafter.

Rheological properties were evaluated at 25 °C by means of a rotational rheometer (Modular Compact Rheometer MCR302, Anton Paar GmbH, Graz, Austria), equipped with a cone plate (C50/1: Ø 50 mm; angle = 1°) combination as measuring system.

Two kinds of rheological measures were carried out to compare formulations: oscillatory and viscosity tests.

Oscillatorytests (strain sweep and oscillation test) were carried out. At first, a strain sweep test enabled finding the linear viscoelastic region; subsequently, a stress chosen in the linear viscoelastic region was applied at frequencies in the range of 0.1–100 rad/s, corresponding to human knee joint solicitations, to measure the viscoelastic moduli (G’ = elastic modulus and G’ = viscous modulus) versus frequency profiles.

A dynamic viscosity test was also effected in the shear rate range 0.01–300 s^−1^.

### 2.5. Optimization Procedure

The following response variables were considered for each formulation: the frequency and the viscosity measured at the cross-over point (i.e., when G’ = G’’), the tan δ (i.e., G’’/G’ ratio) calculated at 2.5 Hz, which was chosen as the frequency exerted on knee joints during a run and the dynamic viscosity measured at 0.01 s^−1^ shear rate, which was chosen to mimic in vivo rest conditions.

The relationship of each response variable with the two factors (polymer concentration and relative fraction of the two HA grades) was investigated. Experimental data were subjected to multiple regression analysis, effected by means of a statistical software package (Statgraphics 5.0, Statistical Graphics, Rockville, MD, USA) [19,20,21].

A series of models including linear, quadratic, and special cubic was considered.

The best-fit model was chosen on the basis of statistical parameters such as F-ratio for significance of regression and adjusted correlation coefficient for the goodness of fit of the model [21,22,23].

The effect of each factor and of their interactions on each response variable was estimated by means of Pareto charts. Two-dimensional (2D) contour plots showing the iso-response lines of each response variable as a function of the levels of two factors were obtained.

### 2.6. Preparation and Characterization of Optimized HA Formulation

The formulation of optimized composition, chosen on the basis of the results of the experimental design, was prepared as described in Section 2.3 and subjected to the same characterization carried out on the nine formulations of the full factorial design.

In addition, syringeability measures were carried out by a dynamometer (Mod. 5942-INSTRON^®^, Norwood, MA, USA) equipped with a syringe text fixture able to accommodate a wide variety of syringe sizes. The syringe test fixture complies with the ISO 7886-1 Annex G (Test method for forces required to operate plunger). During the test, a compression rod, mounted at the crosshead, moves down at constant velocity and pushes the plunger, ejecting the product through a 27 G needle (0.4 × 40 mm) (Terumo, Leuven, Belgium).

A run speed of 10 mm/min was set to simulate the injection rate during product administration. Force versus time data were recorded, and the maximum force required was retained as extrusion force.

### 2.7. PC Emulsion Preparation

Injectable grade fat-free soybean phospholipid rich in PC is not soluble, but it is dispersible in water to form an oil/water emulsion. Preliminary studies proved that the maximum PC concentration suitable for stable o/w emulsion was 0.05% w/v (data not shown); this concentration is half that of PC in healthy synovial fluid (≅ 0.1% w/v).

The PC o/w emulsion was prepared in two steps: at first, PC was dispersed in water under magnetic stirring (C-MAG HS 7, IKA^®^-Werke GmbH & Co. KG, Staufen, Germany) at 1000 rpm for 10 minutes; then, the dispersion was homogenized with a state rotor system (Ultraturrax T10, IKA^®^-Werke GmbH & Co. KG, Staufen, Germany) at 30,000 rpm for 5 minutes. The mean particle size of PC droplets was 219.9 nm and the PDI was 0.397 (Zetasizer Nano ZS, Malvern Instruments Ltd., Worcestershire, UK).

### 2.8. Preparation of the Optimized HA + PC Formulation

The two liquid phases were separately prepared. The first phase, prepared according to the procedure described in Section 2.3, consisted in the HA optimized formulation 10% more concentrated than that expected in the final product: namely, the total HA concentration was equal to 1.65% w/v, corresponding to 0.96% w/v of VHMW HA grade and 0.69% w/v of LMW HA grade.

The second phase consisted of a 0.5% w/v PC o/w emulsion, which was prepared according to Section 2.7. The second phase was added to the first one in a 1:9 volume ratio. The two phases were mixed for 1 hour at 300 rpm by means of mechanical stirring with a paddle (Eurostar 20, IKA^®^-Werke GmbH & Co. KG, Staufen, Germany) to obtain a new and homogeneous liquid phase that represented the desired HA + PC formulation.

In order to exclude foreign particles, the emulsion was filtered under pressure through a polypropylene filter with 5 µm pores (Sartopure PP2 MidiCap, Sartorius Stedim Biotech GmbH, Gottingen, Germany). The filtrate was left to rest overnight to eliminate air bubbles. The day after, the bulk solution was divided into pre-filled syringes that were steam sterilized in autoclave (FOB2-TS, Fedegari, Pavia, Italy) at 121 °C for 15 min.

### 2.9. Characterization of the Optimized HA + PC Formulation

An optimized HA + PC formulation was characterized for pH, osmolality, rheological properties, as described for HA-based formulation, and tribology measures.

#### 2.9.1. Tribology Measures

Tribology measures were performed using a rotational rheometer (Modular Compact Rheometer MCR302, Anton Paar GmbH, Graz, Austria) equipped with the tribological measuring cell T-PTD 200.

To best simulate in vivo conditions, a biological substrate (pig femur) was used.

The formulation under test (0.5 g) was loaded on the cartilage placed on a steel disc and a probe, containing a cylinder of bone plus cartilage, was lowered to contact the sample, exerting a force equal to 1 Newton. To minimize sample evaporation, measures were effected at 20 °C. Thermosetting was assured by a Peltier heating plane.

The lubrication properties of the optimized HA + PC formulation was compared with those of the optimized HA formulation. Physiologic solution was also subjected to tribology measures as control.

#### 2.9.2. Stability Studies

A preliminary stability study was carried out in ICH long-term (25 °C/60% RH) and accelerated (40 °C/75% RH) conditions on the optimized HA + PC formulation, which was packaged in pre-filled syringes. The parameters taken into account at each and every checkpoint (1, 2, 3, and 6 months) and compared with those measured at time zero were frequency at cross over, dynamic viscosity in rest condition (0.01 s^−1^), pH, and osmolality.

#### 2.9.3. Statistical Analysis

Whenever appropriate, experimental values of the various types of measures were subjected to statistical analysis. In particular, one-way Anova, followed by post hoc Sheffè test or Mann–Whitney U-test were employed (Statgraphics 5.0, Statistical Graphics Corporation, Rockvillle, MD, USA) [24].

## 3. Results and Discussion

### 3.1. Properties of the Formulations of the Full Factorial Design

**Figure 1:**
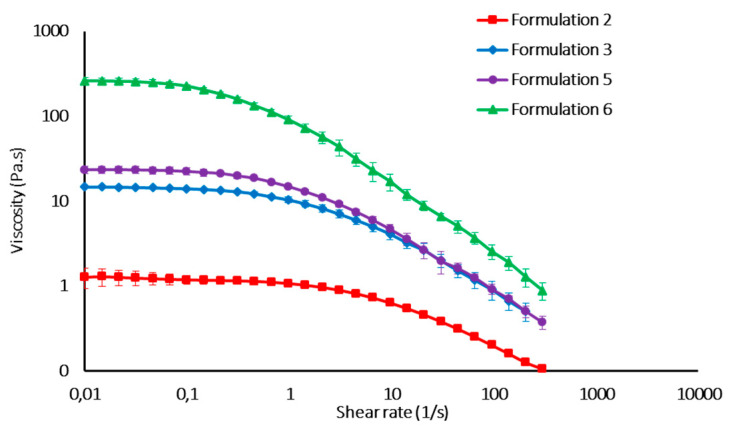
Flow curves of some HA formulations of the experimental design (mean values ± s.d., *n* = 3).

**Figure 2:**
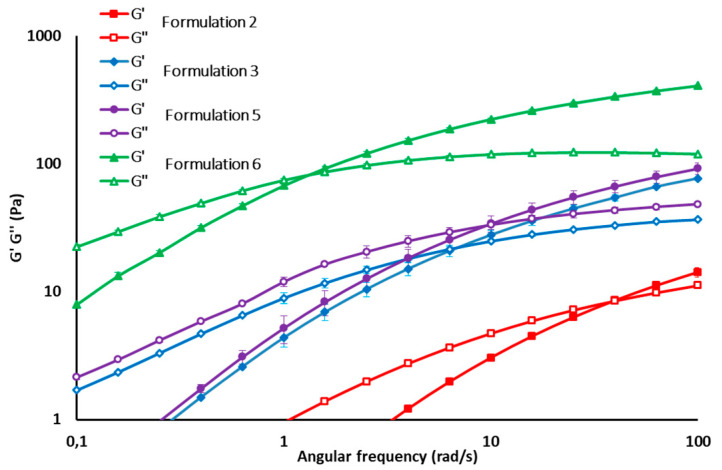
G’ and G’’ profiles of some HA formulations of the experimental design (mean values ± s.d., *n* = 3).

All formulations of the experimental design were characterized by pH value in the range 6.5–7.0 and osmolality was in the range 250–400 mOsm/kg, which is in the range of an intra-articular environment in physiologic conditions. Dynamic viscosity measures demonstrated that all formulations considered in the experimental design are characterized by pseudoplastic behavior, which is functional to easy syringeability. As an example, the flow curves and G’ and G’’ profiles of some formulations (2, 3, 5, 6) are reported in Figure 1 and Figure 2, respectively.

The mean values of all the response variables for the experimental design are reported in Table 3 together with those of the healthy synovial fluid [10], which was taken as a reference.

It can be observed that formulations 1, 4, and 7, containing a high fraction of LMW HA, are characterized by poor viscoelastic properties with a prevalence of viscous over elastic behavior, as demonstrated by the tan δ values, which are much higher than those of synovial fluid in physiologic conditions. Formulation (9) is not suitable, as well, for intra-articular administration, since the high fraction (9:1) and concentration (2.7% w/v) of VHMW HA determine the high rigidity of the system, as demonstrated by the high viscosity at cross over and high viscosity at 0.01 s^−1^ shear rate. All the other formulations, with the exception of formulation 2, are characterized by frequency and viscosity values at cross over in the range of those reported for healthy synovial fluid.

A low VHMW HA fraction always determines an increase in tan δ values, as demonstrated in the case of Formulations (1), (4), and (7) for which the high/low MW ratio is 1:9 and for Formulation (2), for which the concentration of VHMW HA is only 0.5% w/v.

As for dynamic viscosity at a 0.01 s^−1^ shear rate, the majority of formulations are characterized by values similar to that of the healthy synovial fluid, with the exception of formulations 6, 8, and 9 containing the highest concentrations of VHMW HA, which are equal to 1.8%, 1.5%, and 2.7% w/v, respectively.

### 3.2. Multiple Regression Analysis

The best-fit model for all the response variables (frequency and viscosity at cross-over point (G’ = G’’), tan δ at 2.5 Hz, and dynamic viscosity at 0.01 s^−1^ of the nine formulations considered in the experimental design was found to be the quadratic one:

Y_1_ = β_0_ + β_1_X_1_ + β_2_X_2_ + β_3_X_1_X_2_ + β_4_X_1_^2^ + β_5_X_2_^2^ (1)

where Y_1_ = response variable; X_1_ = polymer concentration (factor A); X_2_ = relative fraction of the two HA grades (factor B); β_0_, β_1_, β_2_, β_3_, β_4_, and β_5_ = estimated coefficients of the model.

The equation represents the quantitative effect of factors (X_1_, X_2_) upon each of the responses.

**Figure 3:**
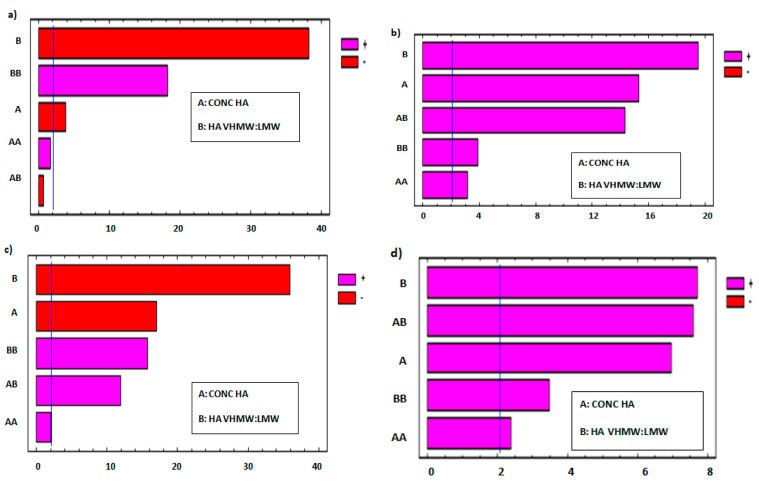
Standardized Pareto chart of the effect of each factor and that of their interactions on each response variable of the full factorial design: (**a**) frequency at cross over, (**b**) viscosity at cross over, (**c**) tan δ at 2.5 Hz, (**d**) dynamic viscosity in rest condition (0.01 s^−1^ shear rate). A = HA concentration; B = VHMW:LMV HA ratio; AA, BB, AB: interaction terms [25].

The effect of each factor and that of their interactions on each response variable was estimated by means of Pareto charts (Figure 3). These graphical representations are useful to determine which factors and relevant interactions have a significant effect, negative or positive, on each response variable of the experimental design [19,20].

In Figure 3a,c, Pareto charts show that both factors exert a negative effect on frequency at cross over and tan δ at 2.5 Hz: in fact, these parameters decrease on increasing polymer concentration and VHMW:LMW HA weight ratio. In particular, tan δ decreases on increasing the VHMW HA, indicating that a higher fraction of the high MW polymer grade determines an increase in the elastic over viscous behavior of the formulation.

The Pareto charts reported in Figure 3b,d demonstrate that both factors have a positive effect on viscosity at cross over and dynamic viscosity in rest condition (shear rate 0.01 s^−1^): in fact, the higher values of both response variables are encountered on increasing the polymer concentration and VHMW: LMW HA weight ratio.

**Figure 4:**
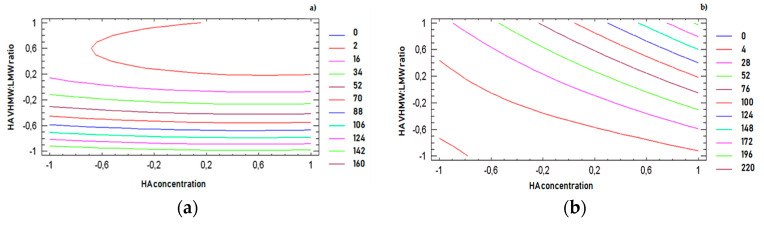

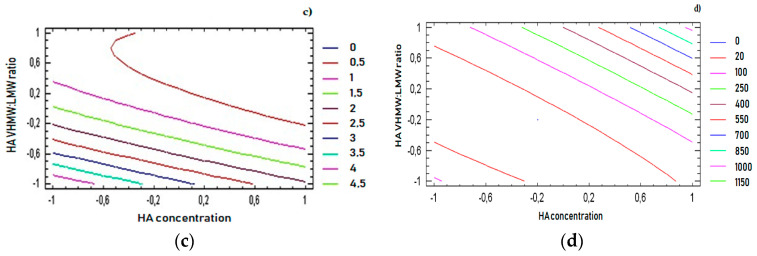
Two-dimensional (2D) contour plots drawn according to the best-fit model for each response variable: (**a**) frequency at cross over, (**b**) viscosity at cross over, (**c**) tan δ at 2.5 Hz, (**d**) dynamic viscosity in rest condition (0.01 s^−1^ shear rate).

For all response variables, 2D contour plots were drawn according to the best-fit model, as illustrated in Figure 4a–d. The lines in each plot represent the formulation compositions for which a same response value is predicted by the model.

**Figure 5:**
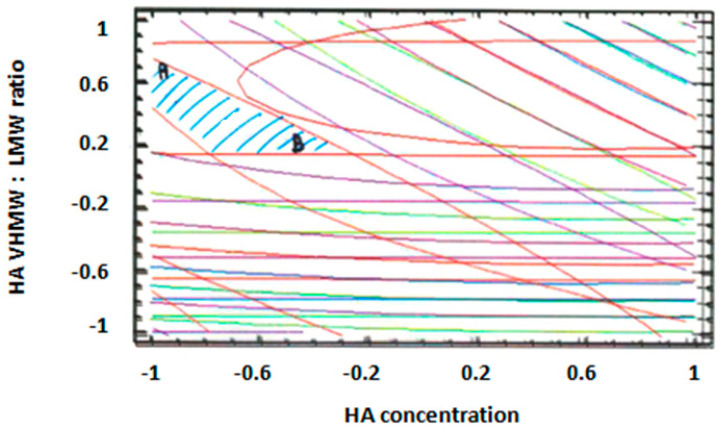
Combined contour plot showing the region of the experimental domain (shaded area) that satisfies all the constraints of the response variables.

The individual contour plots were subsequently superimposed (Figure 5) to identify the region of the experimental domain (shaded area) that fulfills all the constraints decided to optimize the formulation: frequency cross over lower than 20 (rad/s), viscosity cross over in the range 20–80 (Pa.s), tan δ at 2.5 Hz lower than 0.7, dynamic viscosity at 0.01 s^−1^ lower than 100 (Pa.s).

Two optimized formulations were chosen inside the region of the experimental domain satisfying all the constraints. The two formulations (A and B) were selected at the extreme of the optimized area, in order to challenge the model and test its predictive power.

Formulation A contained 1.1% (w/v) of HA with a VHMW:LMW HA ratio equal to 78:22, whereas formulation B contained 1.5% (w/v) of HA with a VHMW:LMW HA ratio equal to 58:42. The % (w/v) composition of the optimized formulations are reported in Table 4.

The results obtained from the characterization of the two optimized formulations are reported in Table 5.

As for rheological properties, the experimental results of the 4 parameters considered fell inside the confidence interval of the values predicted by the model at *p* < 0.05 to indicate its predictive power. pH and osmolality values of both formulations fell inside the acceptance range, and extrusion force was compatible with an easy syringeability. It must be underlined that no statistical difference was observed between homogeneous parameters of the two formulations (Mann–Whitney U-test, N.S.).

On the basis of these results, formulation B was chosen as the optimized HA formulation, since it contains the higher HA concentration (1.5% w/v) and was considered for further steps of pharmaceutical development.

### 3.3. Properties of the Optimized HA + PC Formulation

In Table 6, the aspect, pH, and osmolality values of the optimized HA formulation and of the HA + PC formulation as well as their functional properties (rheological parameters) are compared with those of synovial fluid. It can be observed that the addition of PC does not affect the viscous and viscoelastic properties of the formulation, as confirmed by the statistical analysis (Mann–Whitney test, N.S.), which are comparable to those of the optimized HA formulation. Only the aspect is different: the presence of the water-insoluble PC produces a whitish and opalescent aspect.

**Figure 6:**
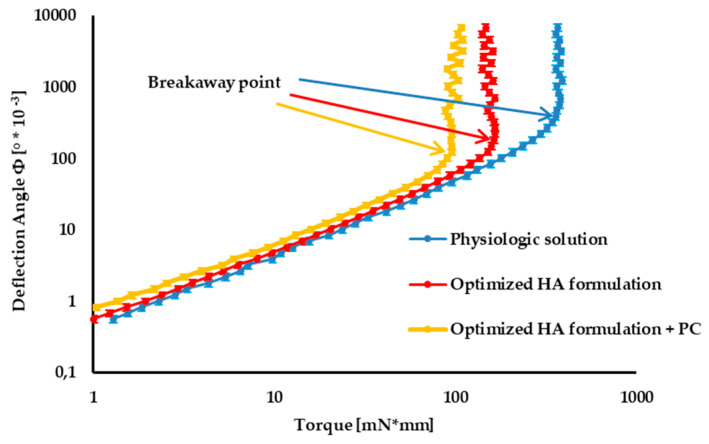
Comparison of tribology profiles (mean values ± s.d., *n* = 3).

In Figure 6, the tribology profiles of the optimized 1.5% w/v HA formulation (formulation B) and the optimized HA formulation containing 0.05 w/v PC are compared with those of physiological solution (taken as control).

In the first portion of the curve, corresponding to low torque values, friction forces between cartilage and formulation prevail over the torque applied: the sample undergoes deformation, but it does not move. At the breakaway point, the slope of the curve dramatically changes: the sample begins to move, since the torque applied prevails over frictional forces.

The smaller the deflection angle at the breakaway point, the greater the lubrication properties of the formulation [26].

The optimized mixture of two HA grades, characterized by quite different MWs, is characterized by much better lubrication properties than the control, as indicated by the markedly smaller deflection angle at the breakaway point.

The lubrication properties of the optimized HA formulation are further improved by the addition of PC, even at low concentration (0.05% w/v), corresponding to half the physiological one (≅0.1% w/v).

### 3.4. Stability Studies

The results of the stability study are reported in Table 7.

As for the aspect, no difference was observed at the various checkpoints with respect to time zero in both storage conditions. This result indicates the physical stability of PC in the oil/water emulsion.

Frequency values at the cross-over point were not affected by increasing the time in both storage conditions in comparison with time zero (one-way Anova, N.S.), thus indicating the maintenance of viscoelastic properties of the formulation.

In the case of dynamic viscosity in rest conditions, no differences were observed between the results obtained in long-term conditions (one-way Anova, N.S.). On the contrary, in accelerated conditions, viscosity at 0.01 s^−1^ tends to slightly decrease over time in comparison with time zero; the statistical analysis pointed out that such a decrease is statistically significant only after 6 months (one-way Anova, *p* < 0.05, post hoc Sheffè test *p* < 0.01). However, it must be underlined that the viscosity measured at 0.01 s^−1^, even in accelerated stability conditions, remains well inside the range (1‒40 Pa.s) reported for healthy synovial fluid.

No statistically significant differences were found for pH and osmolality values in both storage conditions with respect to time zero (One way Anova, N.S.).

## 4. Conclusions

The employment of a very high MW HA in association with a low MW HA represents a winning strategy to prepare a novel HA-based sterile injectable formulation intended for intra-articular administration. The DoE approach proved successful to optimize the best polymer concentration and relative fraction of the two HA grades, saving time and experiments.

The addition of PC to the HA optimized formulation proved useful to obtain a product more similar in composition to the synovial fluid and characterized by lubrication properties markedly improved in comparison with the optimized HA formulation.

## Figures and Tables

**Table 1:** Experimental points of the full factorial design. HA: hyaluronic acid, LMW: low molecular weight, VHMW: very high molecular weight.

Factor		HA Concentration (% w/v)		
		1	2	3
VHMW: LMW HA ratio	1:9	−1; −1	−1; 0	−1; +1
	5:5	0; −1	0; 0	0; +1
	9:1	+1; −1	+1; 0	+1; +1

**Table 2:** % (w/v) composition of the formulations of the full factorial design.

Formulation	HA VHMW	HA LMW	NaCl	Phosphate Buffer pH 7.4
1	0.1	0.9	0.75	up to 100
2	0.5	0.5	0.75	up to 100
3	0.9	0.1	0.75	up to 100
4	0.2	1.8	0.75	up to 100
5	1.0	1.0	0.75	up to 100
6	1.8	0.2	0.75	up to 100
7	0.3	2.7	0.75	up to 100
8	1.5	1.5	0.75	up to 100
9	2.7	0.3	0.75	up to 100

**Table 3:** Response variables of the formulations of the full factorial design (mean values ± s.d.; *n* = 3).

Formulation	Frequency at Cross over (rad/s)	Viscosity at Cross over (Pa.s)	Tan δ at 2.5 Hz	Dynamic Viscosity at 0.01 s^−1^ (Pa.s)
1	87.3 (±10.06)	0.5 (±0.2)	1.357 (±0.237)	0.72 (±0.06)
2	39.1 (±1.01)	8.45 (±0.11)	1.318 (±0.012)	1.27 (±0.34)
3	7.16 (±1.14)	22.39 (±0.15)	0.785 (±0.038)	14.66 (±0.40)
4	55.0 (±5.0)	1.26 (±0.49)	3.157 (±0.819)	1.77 (±0.12)
5	6.00 (±0.97)	28.52 (±1.24)	0.746 (±0.035)	23.24 (±2.38)
6	1.33 (±0.05)	82.14 (±1.30)	0.468 (±0.004)	259.41 (±24.14)
7	31.83 (±12.04)	2.43 (±0.47)	8.049 (±2.472)	3.00 (±0.11)
8	1.84 (±0.04)	66.88 (±2.37)	0.523 (±0.004)	139.07 (±9.75)
9	0.62 (±0.01)	212.90 (±4.87)	0.369 (±0.005)	1184.93 (±129.09)
Healthy synovial fluid	1-10	20-80	0.39	1-40

**Table 4:** % (w/v) composition of the two optimized formulations.

Formulation	VHMW HA	LMW HA	NaCl	Phosphate Buffer pH 7.4
A	0.86	0.24	0.75	up to 100
B	0.87	0.63	0.75	up to 100

**Table 5:** Experimental parameters of the optimized formulations (mean values ± s.d.; *n* = 4).

Formulation	Frequency Cross over (rad/s)	Viscosity Cross over (Pa.s)	Tan δ at 2.5 Hz	Dynamic Viscosity at 0.01 s^−1^ (Pa.s)	pH	Osmolality (mOsm/Kg)	Extrusion Force (N)
Formulation A	8.32 (±0.32)	20.6 (±0.30)	0.815 (±0.016)	10.98 (±0.44)	6.85 (±0.02)	345 (±2)	10.89 (±0.11)
Formulation B	9.09 (±0.27)	22.9 (±0.30)	0.848 (±0.006)	12.07 (±0.46)	6.83 (±0.02)	351 (±2)	11.25 (±0.08)

**Table 6:** Aspect and functional parameters of the optimized HA formulation with and without phosphatidilcholine (PC) (mean values ± s.d.; *n* = 4).

Sample	Aspect	Frequency at Cross over (rad/s)	Viscosity at Cross over (Pa.s)	Tan δ at 2.5 Hz	Dynamic Viscosity at 0.01 s^−1^ (Pa.s)	pH	Osmolality (mOsm/Kg)
HA 1.5% (w/v)	colorless and transparent	9.09 (±0.27)	22.90 (±0.30)	0.848 (±0.006)	12.07 (±0.46)	6.83 (±0.02)	351 (±2)
HA 1.5% + PC 0.05% (w/v)	whitish, slightly opalescent	9.49 (±0.28)	21.65 (±0.37)	0.862 (±0.007)	11.74 (±0.51)	6.64 (±0.07)	345 (±1)
Healthy synovial fluid	N/A	1-10	20-80	0.39	1-40	N/A	N/A

**Table 7:** Aspect and functional parameters of the optimized HA formulation with PC subjected to stability study in ICH long-term and accelerated conditions (mean values ± s.d.; *n* = 3).

Check- Points	Aspect	Frequency Cross over (rad/s)		Dynamic Viscosity at 0.01 s^−1^ (Pa.s)		pH		Osmolality (mOsm/kg)	
		25 °C/ 60% RH	40 °C/ 75% RH	25 °C/ 60% RH	40 °C/ 75% RH	25 °C/ 60% RH	40 °C/ 75% RH	25 °C/ 60% RH	40 °C/ 75% RH
time zero	whitish, slightly opalescent	9.49 (±0.28)		11.74 (±0.51)		6.64 (±0.07)		345 (±1.34)	
1 month	whitish, slightly opalescent	9.55 (±0.32)	10.21 (±0.58)	11.57 (±0.32)	11.03 (±0.41)	6.62 (±0.04)	6.60 (±0.05)	342 (±1.17)	343 (±1.23)
2 months	whitish, slightly opalescent	9.60 (±0.29)	10.26 (±0.69)	11.39 (±0.39)	10.75 (±0.40)	6.62 (±0.03)	6.61 (±0.04)	343 (±1.11)	344 (±1.32)
3 months	whitish, slightly opalescent	9.64 (±0.33)	10.20 (±0.39)	11.21 (±0.31)	9.95 (±0.35)	6.60 (±0.07)	6.59 (±0.05)	347 (±1.30)	344 (±1.21)
6 months	whitish, slightly opalescent	9.77 (±0.47)	10.01 (±0.42)	10.68 (±0.42)	9.77 (±0.39)	6.63 (±0.04)	6.62 (±0.06)	346 (±1.18)	340 (±1.04)
